# Sulfate Availability and Hormonal Signaling in the Coordination of Plant Growth and Development

**DOI:** 10.3390/ijms25073978

**Published:** 2024-04-03

**Authors:** Anna Wawrzyńska, Agnieszka Sirko

**Affiliations:** Laboratory of Plant Protein Homeostasis, Institute of Biochemistry and Biophysics, Polish Academy of Sciences, ul. Pawińskiego 5A, 02-106 Warsaw, Poland; asirko@ibb.waw.pl

**Keywords:** Arabidopsis, phytohormones, plant response, sulfate deficiency, sulfur metabolism

## Abstract

Sulfur (S), one of the crucial macronutrients, plays a pivotal role in fundamental plant processes and the regulation of diverse metabolic pathways. Additionally, it has a major function in plant protection against adverse conditions by enhancing tolerance, often interacting with other molecules to counteract stresses. Despite its significance, a thorough comprehension of how plants regulate S nutrition and particularly the involvement of phytohormones in this process remains elusive. Phytohormone signaling pathways crosstalk to modulate growth and developmental programs in a multifactorial manner. Additionally, S availability regulates the growth and development of plants through molecular mechanisms intertwined with phytohormone signaling pathways. Conversely, many phytohormones influence or alter S metabolism within interconnected pathways. S metabolism is closely associated with phytohormones such as abscisic acid (ABA), auxin (AUX), brassinosteroids (BR), cytokinins (CK), ethylene (ET), gibberellic acid (GA), jasmonic acid (JA), salicylic acid (SA), and strigolactones (SL). This review provides a summary of the research concerning the impact of phytohormones on S metabolism and, conversely, how S availability affects hormonal signaling. Although numerous molecular details are yet to be fully understood, several core signaling components have been identified at the crossroads of S and major phytohormonal pathways.

## 1. Introduction

**Figure 1:**
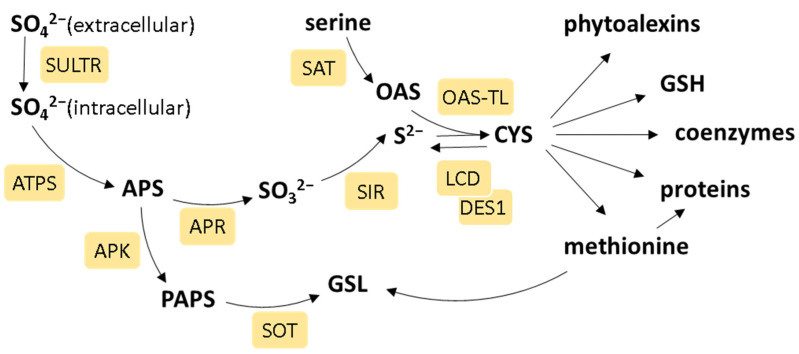
The scheme of plant sulfate assimilation. The enzymes of S metabolism are in light-yellow rectangles and are as follows: sulfate transporter (SULTR), ATP sulfurylase (ATPS), adenosine 5′-phosphosulfate reductase (APR), sulfite reductase (SIR), serine acetyltransferase (SAT), O-acetyl-thiol-lyase (OAS-TL), cysteine desulfhydrase (LCD, DES), APS kinase (APK), and sulfotransferase (SOT). The metabolites in the scheme are as follows: 5′-phosphosulfate (APS), 3′-phosphoadenylylsulfate (PAPS), O-acetylserine (OAS), cysteine (CYS), glutathione (GSH), and glucosinolates (GSLs). Refer to the text for a detailed description.

Plants, being immobile organisms, have developed unique regulatory mechanisms to adjust their growth and development in response to changing environmental conditions. Nutrients serve as crucial signals for plants, transmitting information about the environment to modulate internal programs governing growth and development. The soil exhibits substantial fluctuations in nutrient availability, even over short distances. Consequently, plants have evolved mechanisms to adapt to the diverse nutrient supply in natural environments. Sulfur (S) is an essential element playing a vital role in various physiological processes. S is primarily absorbed in the form of sulfate from the soil by specialized sulfate transporter proteins (SULTRs) [1] (Figure 1). Subsequently, it undergoes translocation to plastids in leaves, also via SULTR transporters, where it is activated to generate adenosine 5′-phosphosulfate (APS), in a process facilitated by ATP sulfurylase [2]. APS is reduced by APS reductase to sulfite, which is then reduced to sulfide by sulfite reductase (SIR). Alternatively, APS is further activated by APS kinase to form 3′-phosphoadenylylsulfate (PAPS), which is necessary for various sulfation reactions in secondary metabolism [3]. Sulfide is incorporated into the carbon skeleton of O-acetylserine (OAS) through the activity of serine acetyltransferases (SATs) and O-acetylserine(thiol)lyase (OASTL), resulting in the production of cysteine [4]. Cysteine is the first S-containing organic compound and a central hub for the synthesis of other compounds, like methionine, sulfolipides, vitamins, coenzymes, and prosthetic groups (iron–S clusters, thiamine, lipoic acid, coenzyme A, etc.) [5,6]. The smallest S-containing molecule is hydrogen sulfide (H_2_S), which is a gasotransmitter regulating many critical processes due to its high chemical reactivity [7]. H_2_S production occurs mainly in chloroplasts thanks to SIR activity; however, it can also be synthesized in mitochondria by cyanoalanine synthase and in cytosol by L-cysteine desulfhydrase (LCD and DES1) [8]. Metabolites associated with stress resistance in plants, such as glutathione (GSH), phytochelatins, defense peptides, and glucosinolates, also contain S [9,10]. GSH is a major cell redox status regulator, and it serves as the primary means of storing and transporting organic S in plants [6]. The circulation of S between plants and the environment is of great importance for the nutrition and health of humans and animals because animals cannot synthesize S-containing amino acids on their own. They rely on obtaining them through their diet, emphasizing the crucial interplay between plants and the environment for overall nutritional well-being [11]. 

**Figure 2:**
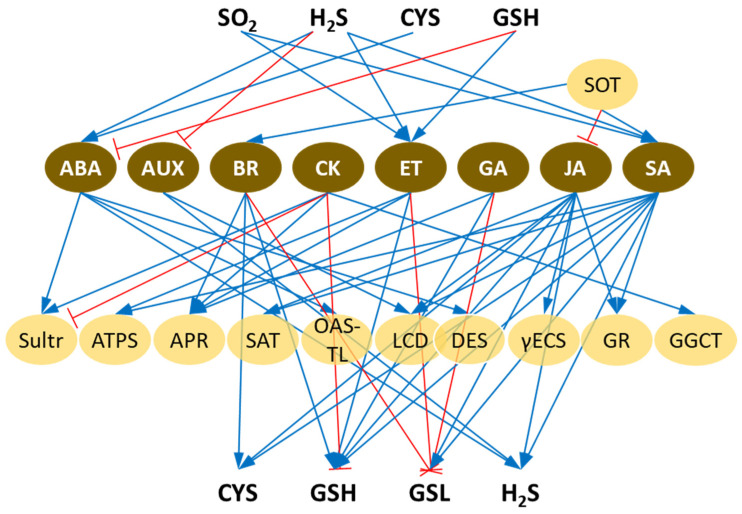
The interplay of sulfur (S) and phytohormones. The enzymes of S metabolism are in light-yellow circles and are as follows: sulfotransferase (SOT), sulfate transporter (Sultr), ATP sulfurylase (ATPS), adenosine 5′-phosphosulfate reductase (APR), serine acetyltransferase (SAT), O-acetyl-thiol-lyase (OAS-TL), cysteine desulfhydrase (LCD, DES), γ-glutamylcysteine synthetase (γECS), glutathione reductase (GR), and γ-glutamylcyclotransferase (GGCT). The phytohormones are in brown circles and are as follows: abscisic acid (ABA), auxin (AUX), brassinosteroids (BR), cytokinins (CK), ethylene (ET), gibberellic acid (GA), jasmonic acid (JA), and salicylic acid (SA). The S-containing metabolites that either regulate phytohormone levels or are affected by them are as follows: sulfur dioxide (SO_2_), hydrogen sulfide (H_2_S), cysteine (CYS), glutathione (GSH), and glucosinolates (GSLs). Positive and negative effects are indicated by blue and red lines, respectively. Refer to the text for a detailed description.

Phytohormones, also known as plant hormones or plant growth regulators, are chemical messengers that play crucial roles in regulating various physiological processes in plants. In contrast to plant growth regulators that are synthetic, phytohormones are natural regulators, which are produced by the plant itself. Phytohormones help coordinate growth, development, and responses to environmental stimuli. They often interact in complex signaling networks, and their levels and activities are finely tuned to orchestrate various aspects of plant growth. The efficient use of nutrients is intricately connected to the signaling pathways of phytohormones, and therefore, they play a crucial role in regulating the assimilation, transport, and metabolism of S. On the other hand, some of the phytohormones require S-containing compounds for their synthesis. The network of mutual relations between phytohormones and S metabolism, according to the current literature, is the subject of this review and is depicted in Figure 2.

## 2. Abscisic Acid Crosstalk with S Metabolism

**Figure 3:**
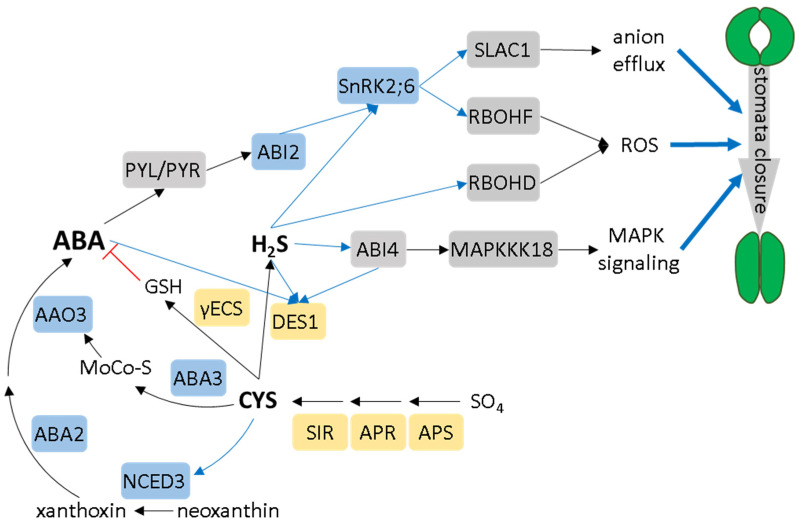
Model depicting the role of sulfur (S) metabolism in abscisic acid (ABA) biosynthesis and the regulation of stomatal closure. Enzymes catalyzing reactions (black arrows) in S metabolism are shown in yellow boxes (ATP sulfurylase (APS), adenosine 5′-phosphosulfate reductase (APR), sulfite reductase (SIR), cysteine desulfhydrase (DES), γ-glutamylcysteine synthetase (γECS)), while enzymes of ABA synthesis and signaling for stomatal closure are shown in blue boxes. The stimulating effects of metabolites or enzymes on downstream reactions are depicted as blue arrows, while red ones indicate a negative impact. Cysteine (CYS) synthesis is limited by sulfate availability, and it affects abscisic aldehyde oxidase (AAO3) activity, serving as the S donor to its cofactor (MoCo-S) in the reaction catalyzed by a sulfurase called ABA3. Moreover, elevated levels of cysteine boost the transcription of 9-cis-epoxycarotenoid dioxygenase 3 (NCED3), thereby augmenting the availability of the substrate precursor for AAO3. PYR/PYL acts as an ABA receptor and regulates ABI2 phosphatase activity, which, in turn, activates SnRK2;6 kinase. SnRK2;6 phosphorylates RBOHF oxidase and activates the anion transporter SLAC1 by phosphorylation at multiple residues [13]. H_2_S produced from cysteine is needed for protein persulfhydration, positively affecting their activity (RBOHD oxidase and ABA-INSENSITIVE 4 transcription factor (ABI4)). Oxidative burst and ROS production, together with anion efflux, are necessary for stomatal closure.

Abscisic acid (ABA), recognized as a stress hormone, plays a crucial role in regulating various physiological processes, including cell division, seed germination, organ senescence, stomatal movement, and the response and adaptation to environmental stress [12]. The synthesis of ABA is connected with S availability. S deficiency results in a reduced rate of cysteine synthesis, subsequently leading to lower levels of ABA and diminished tolerance to abiotic stress [13]. Moreover, sulfate has been shown to impact ABA signaling from roots to shoots, influencing stomatal closure during drought conditions [14]. However, the association between ABA synthesis and S assimilation is somewhat indirect. Cysteine availability plays a crucial role in ensuring an adequate supply of a coenzyme for abscisic aldehyde oxidase, AAO3 [15] (Figure 2). This enzyme requires a molybdenum cofactor that has undergone sulfurylation, a process carried out by a specific sulfurase called ABA3, where cysteine serves as the S donor. ABA3, in turn, activates AAO3, which catalyzes the final step in ABA biosynthesis [16]. Additionally, increased sulfate and cysteine levels enhance the transcription of 9-cis-epoxycarotenoid dioxygenase 3 (*NCED3*), which provides a substrate precursor for AAO3, thus also contributing to ABA biosynthesis [14] (Figure 3). 

The upregulation of chloroplast sulfate transporter *SULTR3;1* transcription in roots under exogenous ABA application suggests its essential role in the coregulation of plastid sulfate uptake [17]. The loss of sulfate transporter3;1 function in *sultr3;1* mutant led to notable reductions in both AAO3 activity and ABA levels in seedlings and seeds [15]. In a *sultr3* quintuple mutant, with the complete knockout of all five members of the subfamily of chloroplast sulfate transporters, the levels of cysteine and ABA were reduced by ∼67 and ∼20% from the wild-type levels, respectively, and this led to the abolishment of stomatal closure [18]. The seed germination of the *sultr3* quintuple mutant displays heightened sensitivity to external ABA and salt stress, yet supplementation with sulfide restores its normal germination rate. In certain situations, particularly during salt and drought stress, ABA is important in signaling and has a substantial influence on sulfate assimilation. These conditions are often accompanied by an increased production of reactive oxygen species (ROS), leading to a reduced S pool and an elevated demand for GSH. Notably, various enzymes involved in sulfate assimilation, such as APS reductase, ATP sulfurylase, sulfite reductase, the cytosolic isoform of O-acetylo-thiolyase, and sulfate transporters (SULTR1;2, 3;1, 3;4, and 4;1), are induced by salt stress [15,19,20]. Surprisingly, this transcriptional regulation does not involve the same mechanisms as salt stress signaling, which includes both ABA-dependent and ABA-independent pathways [21]. While O-acetylo-thiol-lyase is induced by salt in an ABA-dependent manner, the regulation of APS reductase by salt is independent of ABA [20,22].

The widely recognized function of H_2_S is its involvement in the regulation of stomatal opening. This has been thoroughly examined in a recent review [23] and is depicted in Figure 3. The control of stomatal opening exhibits intermittent patterns in mutants lacking the enzyme responsible for H_2_S production—DES1 [24]. Pretreatment with sodium hydrosulfide (NaHS), a H_2_S precursor, not only induced the transcription of ABA receptors during drought stress but also initiated the sulfhydration (or persulfidation, a posttranslational modification) of ABA receptors, namely, Pyrabactin Resistance 1 (PYR1) and Pyrabactin Resistance Like 1 (PYL1) [25]. Likewise, in Arabidopsis *des1* knockout mutants with impaired H_2_S production, this modification was diminished. Also, the persulfidation of SNF1-Related Protein Kinase2.6 (SnRK2.6), which acts as a core component of ABA signaling that controls stomatal movements, was recently reported [26]. The external application of ABA induces the synthesis of H_2_S, indicating the presence of intricate crosstalk between these two signaling molecules under both drought and heat stress conditions [27]. DES1 itself was also shown to be necessary for the expression of several ABA biosynthetic genes, such as *ZEAXANTHIN DEEPOXYDASE* (*ZEP*), *NCED3*, *AAO3*, and *ABA3* [28]. Another study unveiled that ABSCISIC ACID INSENSITIVE 4 (ABI4), a vital transcription factor in the ABA signaling cascade, plays a role in mediating the interaction between ABA and H_2_S at the transcriptional level [29]. ABA accumulation induces a significant production of H_2_S, resulting in the persulfidation of ABI4 at Cys250. This persulfidation enables ABI4 to bind to the promoter of MITOGEN-ACTIVATED PROTEIN KINASE KINASE KINASE 18 (MAPKKK18), which propagates the MAPK signaling cascade induced by ABA [30]. ABI4 also requires persulfidation to bind to the promoter of *DES1* and activate its transcription, thus forming a regulatory loop. This, in turn, contributes to stomatal closure via the ABA-dependent signaling cascade (Figure 3).

Furthermore, stomata are closed in the *cad2* mutant, characterized by reduced GSH levels [31,32]. Decreasing GSH enhanced ABA-induced stomatal closure, while the production of ROS in guard cells was not affected. Thus, GSH is a negative modulator of a signal component other than ROS production in the ABA signal pathway [31]. Another report has shown that this phenomenon is rather connected with the accumulation of cysteine, which is not consumed for GSH production but affects ABA biosynthesis in the *cad2* mutant [13]. On the other hand, the overexpression of the gene encoding γ-glutamylcysteine synthetase, a rate-limiting enzyme in GSH biosynthesis, led to notable decreases in both stomatal aperture and density and, in turn, increased tolerance to drought stress in transgenic Arabidopsis [33]. Therefore, to ensure the proper functioning of ABA, a continuous and steady supply of cysteine is essential. Recently, it was demonstrated that peroxisome-localized sulfite oxidase (SO), which oxidizes excess sulfite to sulfate, has a role in stomatal opening [34]. Sulfite application limited sulfate- and ABA-induced stomatal closure in a SO knockdown Arabidopsis mutant and resulted in significant water loss. At the same time, APS reductase activity was increased, leading to the enhanced production of internal sulfite, further increasing stomatal aperture and water loss. 

The accumulation of ABA induces the negative role of the glutathione S-transferase GSTU17 in stress tolerance by impacting the GSH pool [35]. This suggests a more profound effect of ABA on the control of S metabolism under stress conditions [4]. ABA plays a crucial role in maintaining the redox state by elevating the level of GSH in Arabidopsis [36]. Glutathione peroxidase (GPX) is an antioxidant enzyme utilizing GSH to protect plants from oxidative stress. Arabidopsis *gpx3* mutants, which lack GPX activity, are insensitive to ABA during seed germination due to the modulation of the activity of ABI2 phosphatase [37]. GPX3 physically binds to ABI2 to inactivate it by regulating its redox state. Glutaredoxins (GRXs), thiol-disulfide oxidoreductases that catalyze the reversible reduction of disulfide bonds in proteins using GSH, have been implicated as negative regulators of ABA signaling during seed germination/preharvest sprouting [38]. Most probably, GRXs affect the level of H_2_O_2_, which is known to positively regulate ABA signaling and thereby inhibit seed germination.

## 3. Auxin Crosstalk with S Metabolism

**Figure 4:**
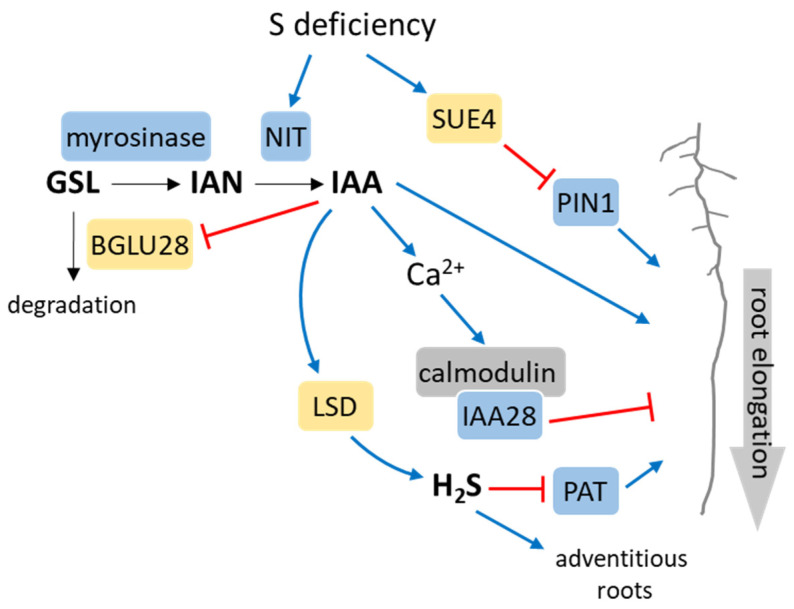
A model linking the response to S deficiency with the auxin role in primary root elongation. In yellow are enzymes of S metabolism: β-glucosidase 28 (BGLU28), L-cysteine desulfhydrase (LSD), and SULFATE UTILIZATION EFFICIENCY 4 (SUE4). In blue are proteins of auxin metabolism: nitrylase (NIT), INDOLE-3-ACETIC ACID INDUCIBLE 28 (IAA28), auxin efflux carrier PIN1 (PIN1), and polar auxin transport (PAT). Other abbreviations are as follows: glucosinolates (GSLs), indole-3-acetonitrile (IAN), and indole-3-acetic acid (IAA). Positive and negative effects are indicated by blue and red lines, respectively. Refer to the text for a detailed description.

Auxins (AUXs) are pivotal plant hormones that exert central control over various aspects of plant growth and development, effectively coordinating responses to diverse environmental conditions [39]. Transcriptome studies have revealed that S deficiency can trigger the expression of genes related to the most common among AUXs—indole-3-acetic acid (IAA) synthesis [40]. This suggests a potential increase in AUX levels, consequently contributing to enhanced root development in Arabidopsis under S-deficient conditions. Glucosinolates, major secondary S metabolites in the *Brassicaceae* family, are decomposed into sulfate and indole-3-acetonitrile (IAN) by myrosinase action [41] (Figure 4). IAN can be converted into IAA under the action of nitrilase (NIT). Three out of the four nitrilases present in Arabidopsis (NIT1, NIT2, and NIT3) can catalyze this reaction [42]. Under S-deficient conditions, the expression of *NIT3* increases, thus promoting the conversion of glucosinolates to IAN and then to IAA. It was therefore believed that the synthesis of AUX is heightened to stimulate root development in Arabidopsis when subjected to S-deficient conditions [43] (Figure 4). However, no disparity in AUX content has been observed in plants growing under S-deficient conditions compared to those in S-sufficient conditions [43]. Other studies have demonstrated that the overexpression of AUX-related genes in response to S deficiency leads to alterations in numerous metabolic processes in plants while not influencing S metabolism [44]. Under the exogenous application of AUX, the S-deficiency-activated expression of β-glucosidase 28 (*BGLU28*), the major catabolic enzyme of glucosinolates, is downregulated [45] (Figure 4). Through the utilization of the DR5::GUS reporter Arabidopsis line responding to AUX levels, it was discovered that the inhibition of lateral root development under S deficiency stems from decreased AUX synthesis or reduced AUX sensitivity. This finding suggests a negative regulatory role for AUX in plants’ response to S deficiency [45]. Hence, it is plausible that AUX regulates root morphology under S-deficient conditions through both positive and negative feedback pathways [46]. The root levels of cysteine, GSH, and IAA exhibit a positive correlation with external sulfate supply within the physiological range, thereby influencing the root system architecture of Arabidopsis plants [47]. Additionally, low sulfate levels lead to the downregulation of genes associated with AUX transport while promoting the accumulation of PLT1 and PLT2 proteins, encoding two AP2 transcription factors essential for root stem cell niche patterning [47]. In a new report, the role of S deficiency-induced SULFATE UTILIZATION EFFICIENCY 4 (SUE4), a novel plasma membrane-localized protein, in primary root elongation is described [48]. The interaction of SUE4 with the polar AUX transporter PIN1 leads to reduced levels of the PIN1 protein, possibly through 26S proteasome-mediated degradation, consequently diminishing AUX transport to the root tips. This process ultimately facilitates primary root elongation (Figure 4).

Alongside the positive regulation pathway, where S deficiency boosts root growth by elevating AUX synthesis, there exists an AUX-related transcription factor IAA28-mediated negative regulation pathway. This pathway serves to impede the augmentation of plant root development under S-deficient conditions, thereby initiating a negative feedback regulation mechanism to restrict root growth [46]. Elevated AUX levels lead to alterations in cell calcium ion concentrations, subsequently upregulating the expression of calmodulin. Calmodulin, in turn, interacts with IAA28, potentially resulting in the inhibition of AUX-induced gene expression [49] (Figure 4). A decrease in GSH levels in the cell (e.g., during S starvation) causes a significant decrease in the AUX gradient in the root tips, leading to alterations in lateral root growth and density [50]. Interestingly, a genetic screen investigating alterations in the S limitation response unveiled that mutants in the *BIG* gene, encoding a protein responsible for the polar transport of AUX, exhibited the constitutive upregulation of genes typically induced by S deficiency [51]. Nevertheless, given that the loss of *BIG* resulted in elevated AUX levels, and considering that AUX treatment also induced these genes even under full-S conditions, these results suggest that BIG might be indirectly related to the sulfate starvation response. The establishment of an AUX gradient, facilitated by polar AUX transport (PAT) from aerial to basal tissues, is closely linked to numerous physiological processes [52]. In Arabidopsis, elevated levels of H_2_S hindered PAT, subsequently leading to modifications in root structure [53]. Moreover, H_2_S disrupted AUX transport by affecting the distribution of PIN proteins, ultimately causing alterations in root development (Figure 4). The localization of PIN proteins relies on actin-dependent mechanisms, and the expression of various actin-binding proteins (ABPs), as well as the AUX receptor, is influenced by H_2_S [54]. It was also observed that IAA had the ability to increase the expression of the L-cysteine desulfhydrase (*LCD*) gene in Arabidopsis seedlings, which stimulated H_2_S biosynthesis and the subsequent development of adventitious roots [55] (Figure 4). These findings indicate that there is reciprocal regulation between H_2_S and AUX in the regulation of root formation.

Moreover, SURE, the key regulatory *cis*-element of the S-deficiency response, includes a binding sequence (GAGACA) for the AUX response factor. Nevertheless, there is currently no evidence suggesting a connection between SURE and AUX signaling [56].

## 4. Brassinosteroid Crosstalk with S Metabolism

Brassinosteroids (BRs) play a crucial role in governing both growth and development [57], but when applied externally, they exhibit the ability to increase oxidative stress tolerance through various mechanisms [58]. Very little is known about the effect of BRs on S metabolism. It was shown that BRs regulate glucosinolate levels in Arabidopsis and radish by increasing the expression of glucosinolate biosynthesis genes [59]. As a result, the Arabidopsis BRASSINOSTEROID INSENSITIVE 1 (*bri1-5*) mutant defective in BR receptor functions is preferred by chewing insects over wild-type plants, because it exhibits an altered glucosinolate profile. Glucosinolates are an important class of secondary metabolites in *Brassicales* plants with a critical role in defense against pathogens and herbivores [60]. In a contradictory report, it was demonstrated that BRs inhibit the accumulation of glucosinolates while simultaneously enhancing the biosynthesis of primary S metabolites, such as cysteine and GSH, in both Arabidopsis and Brassica crops [61]. BRASSINAZOLE RESISTANT 1 (BZR1), a key regulator in BR signaling, exerts specific transcriptional regulation by directly repressing the biosynthesis of indolic glucosinolates through MYB51-dependent mechanisms, while it partially suppresses the biosynthesis of aliphatic glucosinolates via MYB29-dependent pathways. Moreover, through the direct transcriptional activation of two APS reductases, *APR1* and *APR2*, BZR1 increases the biosynthesis of cysteine. This dual effect fine-tunes both secondary and primary S metabolism, ultimately promoting plant growth. Heat stress decreases S content while increasing ATP sulfurylase activity and the contents of cysteine and methionine in rice. However, the application of BRs under heat stress further increases S assimilation [62]. 

BRs undergo modification through sulfation, and two sulfotransferases responsible for this reaction have been characterized [63]. In vitro, the sulfotransferase SOT12 has the capability to sulfate several BRs, yet it demonstrates a distinct preference for the BR precursor 24-epicathasterone [63]. SOT10 exhibits a preference for the biologically active end-products of BR biosynthesis, such as 24-epibrassinolide and naturally occurring (22R,23R)-28-homobrassinosteroids. Notably, the sulfation of 24-epibrassinolide can result in the suppression of its bioactivity [64]. However, BR-related phenotypes were not observed in either *sot10* or *sot12* loss-of-function mutants [65]. The attachment of polar moieties, such as in sulfation, to comparatively non-polar BRs has been suggested as a strategy to enhance the intracellular transport of BRs. This movement is crucial for transferring them from their origin at the endoplasmic reticulum to their site of perception at the plasma membrane [66]. Consequently, it is proposed that SOTs play a role in regulating the activity, mobility, and/or perception of BRs, although the precise mechanism(s) remain elusive. 

## 5. Cytokinin Crosstalk with S Metabolism

Ever since their initial discovery in the last century as the regulators of cell division, cytokinins (CKs) have been associated with numerous physiological processes in plants, including growth and development, as well as diverse responses to environmental stimuli [67]. The connection between S deficiency and CK status is suggested by the downregulation of *IPT3*, encoding isopentenyl transferase, which catalyzes the first rate-limiting step of CK synthesis, in the roots of Arabidopsis plants [68] and changes in CK levels observed in poplar trees [69]. The gene *GGCT2;1*, encoding a crucial enzyme involved in GSH degradation, exhibits high responsiveness to both S starvation and CKs [70]. This indicates that CKs may have a role in regulating GSH homeostasis, and the CK-mediated degradation of GSH could potentially play a significant physiological role in nutrient mobilization. It was shown that CKs inhibit the expression of the major S transporters *Sultr1;1* and *Sultr1;2*, thereby negatively regulating S uptake in Arabidopsis. This process relies on CK receptors CRE1/WOL/AHK4 [71]. Nonetheless, CKs do not impact the induction of S assimilation under S-deficient conditions. This suggests that the negative regulation of CKs and the signaling pathway for S uptake under S-deficient conditions are distinct and independent pathways [71]. Furthermore, research has indicated that the exogenous treatment of Arabidopsis leaves with CKs induces the expression of *Sultr2;2*, a S transporter expressed only in the bundle sheath and veins, and the key S assimilation enzyme APS reductase [72]. The treatment of potato plants with CKs did not affect either the glutathione transferase or glutathione reductase activity, nor did the level of GSH change. However, when potato plants were challenged with salt stress, all these parameters increased, and they were further positively affected by CK application [73]. These findings imply that the precise role of CKs in regulating the plant response to S deficiency is not yet fully comprehended.

A recent study shows that the application of CKs to Arabidopsis resulted in the induction of gene expression patterns typically associated with S starvation, concurrent with a reduction in both sulfate and GSH levels [74]. In contrast, mutants deficient in the CK receptor ARABIDOPSIS HISTIDINE KINASE 3 (AHK3), as well as CK-deficient plants, exhibited an accumulation of GSH. Moreover, CK-deficient plants showed enhanced root growth when exposed to chemicals that deplete GSH levels, indicating a heightened ability to sustain GSH levels in these plants [74]. Thus, CKs emerge as crucial regulators of S uptake, assimilation, and redistribution in plant defense against xenobiotics, as well as in the modulation of root growth. 

## 6. Ethylene Crosstalk with S Metabolism

Ethylene (ET), a gaseous phytohormone, actively participates in various physiological processes, including seed germination, organ maturation and senescence, and stomatal movement, as well as the response and adaptation to environmental stress [75]. The biosynthesis of ET is firmly connected with methionine metabolism [76]. Methionine is activated to form S-adenosylmethionine (SAM), which undergoes transformation to produce 1-aminocyclopropane carboxylate (ACC). ACC serves as the substrate for ET biosynthesis. Prolonged S deficiency leads to a lowering of the ACC pool, thus negatively affecting the ET level. Nonetheless, this is not the only association between ET synthesis and S. During the synthesis of ET from ACC, highly toxic hydrocyanic acid is produced. The primary detoxification mechanism for cyanide involves its reaction with cysteine, a process catalyzed by ß-cyanoalanine synthase, which is a member of the O-acetylserine-thiolyase family [77]. Transgenic *Nicotiana tabacum* plants overexpressing a tomato gene that encodes glutathione synthetase 1 (*GSH1*) significantly upregulated the expression of ET biosynthesis genes, such as ACS (1-aminocyclopropane-1-carboxylate synthase) and ACO (1-aminocyclopropane-1-carboxylate oxidase), when compared to wild-type plants [78]. Similarly, transgenic *A. thaliana* plants overexpressing *GSH1* showed elevated GSH contents and a strong increase in ET biosynthesis transcripts (*ACS*, *ACO*), while the expression of these genes was downregulated in the GSH-depleted *pad2-1* mutant [79]. In addition, S-glutathionylation of the ACO1 protein was detected. Such posttranslational modification may impact protein stability/activity. Another putative link to S metabolism is the interaction of tobacco ACO1 with the UP9C protein of unknown function [80]. UP9C belongs to the plant-specific family of LSU proteins, which are strongly induced at the transcriptional level during S deficiency [81,82]. A short-term S deficiency triggers the increased expression of certain ET-related genes and the accumulation of ET in tobacco, a response that is notably absent in antisense *UP9C* plants [80,83]. This may suggest the relevance of the interaction between UP9C and ACO1 for its proper functioning. 

ET plays a pivotal role during heavy-metal stress. After exposure to cadmium, plants quickly allocate resources to produce phytochelatins, which are oligomers of GSH, leading to the disruption of the redox environment by temporarily reducing GSH concentrations. As a result, a cascade of signaling responses is triggered, with ET playing a crucial role in restoring GSH levels [84]. It was recently shown that there is crosstalk between glucosinolate levels and the expression of ET-related genes under S deprivation [85]. Apparently, the genes involved in the ET response were not regulated by S limitation in a double *bglu28/30* Arabidopsis mutant unable to catabolize glucosinolates. A rapid reduction in glucosinolate levels following ET treatment further elucidated ET’s role in controlling their accumulation [86]. On the other hand, it was shown that ET has a positive effect on the sulfate reduction pathway. It was observed that the levels of *APR1* and *APR3* transcripts, as well as overall APS reductase activity, were increased after the exogenous application of ACC [87]. Additionally, some reports indicate that ET enhances ATP sulfurylase activity and promotes sulfate uptake in *Brassicaceae* plants [88,89]. However, the treatment of oilseed rape plants with ET with prolonged S deprivation has an adverse effect. Through the S-starvation-induced downregulation of ATP sulfurylase and, to a greater extent, sulfate transporter genes, ET could regulate S acquisition [90]. Interestingly, the heterologous expression of the Arabidopsis ET receptor gene, *etr1-1* (though encoding a mutated ETR1 protein incapable of transmitting ET signals post-hormone binding), in *N. attenuate* plants led to a reduction in sulfate uptake and impaired S metabolism [91]. The phenotypes resembling those of plants experiencing S deficiency observed in these seedlings imply that alterations in ET signaling mimic the signal associated with S deficiency. It was reported that ET facilitated the abscission of the petiole in tomatoes and the floral organs in roses, and the external application of H_2_S counteracted these effects by inhibiting the transcription of genes encoding enzymes such as cellulase and polygalacturonase, which are associated with cell wall modification [92]. Moreover, in banana, H_2_S downregulated the expression of *ACS1*, *ACS2*, and *ACO2* while concurrently upregulating the expression of ET receptors, including Ethylene Receptor (*ETR*), Ethylene Response Sensor1 (*ERS1*), and *ERS2* [93]. The analysis of grape berry transcriptomes following treatment with SO_2_ demonstrated significant changes in gene expression profiles, with notable upregulation observed in transcripts associated with AUXs, ET, and jasmonate signaling pathways [94]. 

ETHYLENE INSENSITIVE LIKE 1 (EIL1), one of the crucial signaling factors of the ET pathway, is involved in transcriptional regulation during S deficiency [95]. It is worth mentioning here that SULFUR LIMITATION 1 (SLIM1), belonging to the same EIL protein family, is the pivotal factor in S deficiency signaling. An examination of *slim1* mutants demonstrated that SLIM1 influences the expression of various genes involved in enhancing the flux through the S assimilation pathway, the transport of S to the shoot, and the breakdown of glucosinolates under S-deficient conditions [95,96]. It was clearly demonstrated, though, that SLIM1 does not take part in ET signaling [96].

## 7. Gibberellic Acid Crosstalk with S Metabolism

Gibberellic acid (GA), a naturally occurring tetracyclic diterpenoid plant hormone, is involved in regulating various growth and developmental processes in plants. Furthermore, GA plays a crucial role in alleviating the adverse effects of abiotic stressors [97]. It has been reported that the transcriptional induction of all three isoforms of APS reductase due to salinity can be impeded in a GA-insensitive mutant by disrupting GA signaling [87]. Interestingly, the enzyme activity remains unaltered. In another study, it was proposed that the combined application of GA and S has the potential to alleviate oxidative stress in mustard plants exposed to cadmium stress [98]. This mitigation is attributed to the formation of ET, enhanced S-use efficiency, improved photosynthesis, and increased GSH production. The improved S acquisition upon GA application under cadmium stress helped to boost the photosynthetic performance and growth of mungbean and that involved nitric oxide signaling [99]. GA tightly regulates the process of programmed cell death in cereal aleurone cells occurring after germination [100]. It was shown that the application of SO_2_ alleviates the programmed cell death of GA-treated barley aleurone cells by reducing ROS accumulation by enhancing the activities of antioxidant enzymes [100]. GA was found to exert a negative influence on MYB51 transcription (responsible for glucosinolate synthesis) in emerging true leaves of *Brassica oleracea* [101]. Interestingly, GA along with glucose had a positive effect on glucosinolate accumulation in *Brassica oleracea* sprouts [102]. There is still not enough research on GA and S crosstalk, especially on the molecular mechanisms behind it. 

## 8. Jasmonic Acid Crosstalk with S Metabolism

**Figure 5:**
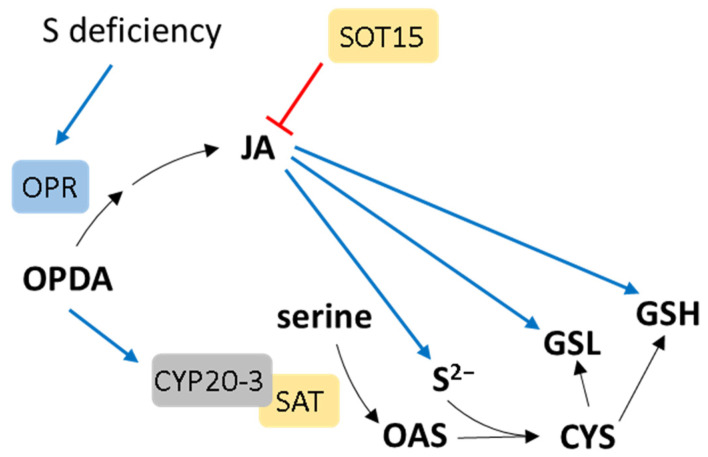
The mutual regulation of S metabolism and jasmonic acid biosynthesis and signaling. S deficiency activates the expression of 12-oxophytodienoate reductase (OPR), leading to higher JA levels. This positively impacts the levels of sulfide, glutathione (GSH), and glucosinolates (GSLs). 12-Oxo-phytodienoic acid (OPDA) binds to cyclophilin CYP20-3, thus stimulating its binding to serine acetyltransferase (SAT) to activate O-acetylserine (OAS) production. The sulfotransferase SOT15 inactivates JA through sulfation. Positive and negative effects are indicated by blue and red lines, respectively.

Jasmonates are derived from the controlled oxygenation of polyunsaturated fatty acids by lipoxygenases. They primarily contribute to stress-related responses by regulating the transcript levels of numerous genes involved in stress tolerance [103]. Jasmonic acid (JA) or its derivatives have been observed to induce the synthesis of sulfide, GSH, and glucosinolates without causing alterations in the steady-state levels of cysteine [104] (Figure 5). Transcription factors such as MYC2, MYC3, and MYC4, which regulate JA responses, are also implicated in the control of glucosinolate synthesis [105]. These findings suggest that JA plays a beneficial role in the regulation of S metabolism. The expression profiling of metabolic genes in Arabidopsis in response to JA revealed that the regulation of genes in primary and secondary S-related pathways is by far the most strongly affected among the genes [106]. Additionally, the synthesis of JA was upregulated as a consequence of oxidative stress induced by a deficiency in GSH. In GSH-deficient *cad2* Arabidopsis mutants, there are observed alterations in the expression of genes related to JA synthesis and activation compared to wild-type plants [107]. Moreover, these researchers discovered that the application of exogenous GSH restores the normal expression of JA-related defense genes in *cad2* mutants. The expression of 12-oxophytodienoate reductase (OPR), encoding an enzyme in the JA synthetic pathway, was increased in response to a transfer to S-deficient conditions [108] (Figure 5). Interestingly, the plastidic cyclophilin CYP20-3 was shown to be a protein able to bind a precursor of JA, 12-oxo-phytodienoic acid (OPDA) [109]. The binding of OPDA to CYP20-3 enables an interaction between the cyclophilin and serine acetyltransferase, the enzyme responsible for synthesizing the cysteine precursor. This process enhances cysteine production, and by subsequently increasing GSH synthesis, it influences the redox potential of the cells [109] (Figure 5). JA is also the substrate of sulfotransferase SOT15, and as such, it is inactive and negatively affects the expression of genes encoding enzymes in JA biosynthesis [110]. Therefore, SOT15 serves as a component of an inhibitory mechanism within the JA signaling pathway, acting as a regulatory “off” switch (Figure 5). In Arabidopsis, JA significantly increased H_2_S content, the activities of L-cysteine desulfhydrase, glutathione reductase, and γ-glutamylcysteine synthetase [111]. Moreover, it has been reported that the interaction between H_2_S and JA regulates various plant functions, such as the induction of stress resistance. As an example, in Arabidopsis cotyledones, H_2_S has been reported to act downstream of JA, inhibiting stomatal development [112]. The high stomatal density of JA-deficient mutants could be rescued by exogenous NaHS treatment. Apart from that, JA stimulates H_2_S generation to improve physiological adaptation to heavy-metal exposure, probably by initiating CDPK signaling [113]. The interaction between H_2_S and JA under various stress conditions has been extensively reviewed by Li et al. [114]. 

## 9. Salicylic Acid Crosstalk with S Metabolism

**Figure 6:**
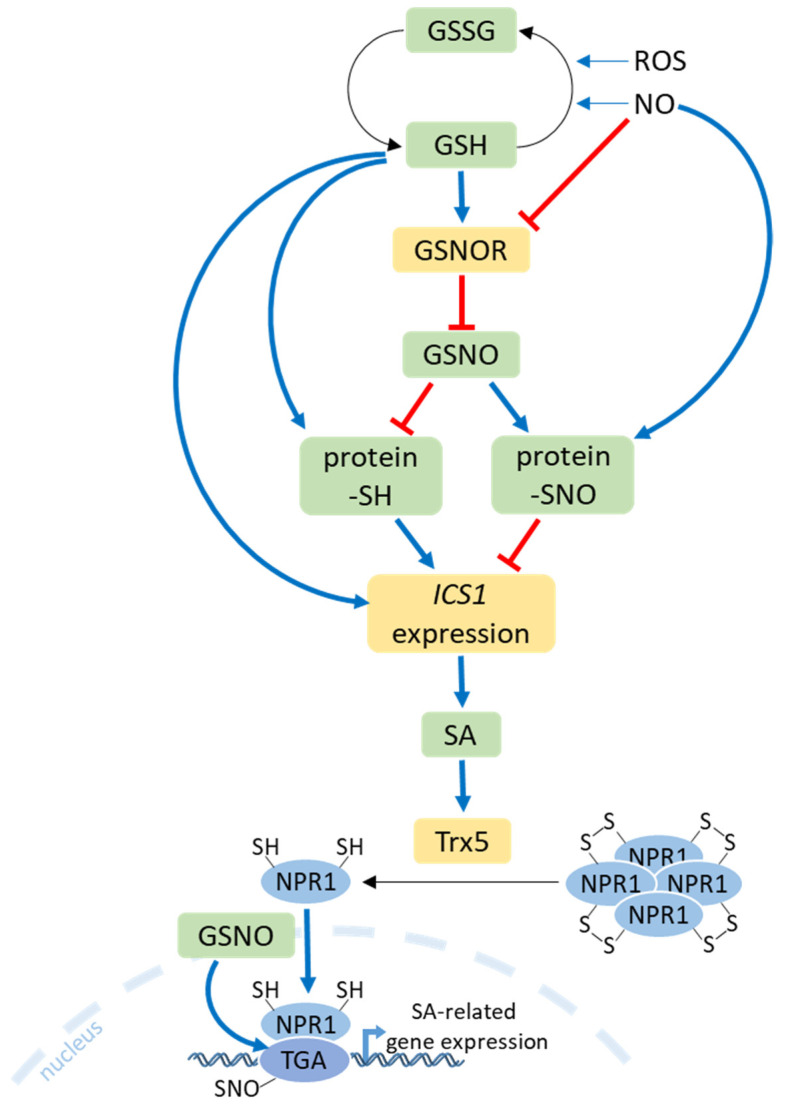
The glutathione (reduced/oxidized form, GSH/GSSG)-mediated regulation of the plant hormone salicylic acid (SA) through the expression of isochorismate synthase (*ICS1*). The generation of reactive oxygen species (ROS) and nitrogen oxide (NO) during plant defense alters the GSH/GSSG ratio. NO inhibits while GSH activates S-nitrosoglutathione reductase (GSNOR), which catalyzes the degradation of S-nitrosoglutathione (GSNO). The breakdown of GSNO results in the decreased formation of protein-SNO, thereby preserving protein-SH groups, which activates the increased expression of *ICS1* and the synthesis of SA. Furthermore, NO accumulating during the initial stages of plant defense can react with GSH to produce GSNO, which, in turn, suppresses the accumulation of SA. The accumulation of SA triggers the expression of defense genes by causing conformational changes in the NON-EXPRESSOR OF PATHOGENESIS-RELATED 1 protein (NPR1). Alterations in the redox state of plant cells cause the reduction of the cysteine residues within NPR1, leading to the release of NPR1 monomers from the tetrameric complex, catalyzed by thioredoxins (TRXs). Activated NPR1 monomers are translocated to the nucleus, mediated by GSNO. The activated NPR1 monomer interacts with TGA transcription factors to induce the expression of SA-related genes, while the S-nitrosylation of TGA, facilitated by GSNO, further increases gene expression. Positive and negative effects are indicated by blue and red lines, respectively.

Salicylic acid (SA) is a regulator of pathogen responses and cell death [115]. A significant interaction exists between SA-mediated S assimilation and stress defense responses. Plants treated with exogenous SA exhibit increased GSH content, accompanied by heightened glutathione reductase activity [116]. SA also increases cysteine content as a result of higher activities of ATP sulfurylase and serine acetyltransferase [117]. It has been reported that the exogenous application of SA under various abiotic stresses in *Brassica napus* leads to an increase in various S-containing secondary metabolites, such as thionines, glucosinolates, and GSH [118]. On the other hand, SA homeostasis is positively regulated by a sulfotransferase (SOT12) via sulfation, which results in higher resistance to pathogen infection [119]. The burst of ROS during pathogen infection leads to alterations in the ratio of reduced glutathione (GSH) to oxidized glutathione (GSSG). This change induces the expression of the isochorismate synthase 1 (*ICS1*) gene, which encodes the key enzyme involved in SA biosynthesis in Arabidopsis [120] (Figure 6). Indeed, studies have demonstrated that increasing the GSH content through the overexpression of tomato *GSH1* in transgenic tobacco leads to enhanced GSH synthesis and higher levels of SA. Consequently, these plants exhibit higher resistance to *Pseudomonas syringae* [78]. Similarly, the infiltration of exogenous GSH into the leaves resulted in a substantial rise in bound SA and, to a lesser extent, free SA levels in tobacco, especially following tobacco mosaic virus (TMV) infection [121]. Also, elevated levels of glutathione in TMV-infected tobacco were observed, and these could compensate for SA deficiency in NahG mutant plants to maintain virus resistance [121]. 

S-nitrosoglutathione (GSNO), formed through the reaction of NO with GSH, serves as a crucial S-nitrosylating agent in plant cells [122]. S-nitrosoglutathione reductase 1 (GSNOR1) catalyzes the degradation of GSNO to GSSG and NH3, utilizing reduced β-nicotinamide adenine dinucleotide (NADH). The loss of GSNOR1 function increased protein-SNO levels in Arabidopsis, leading to enhanced susceptibility to *Pseudomonas syringae*, while increased activity reduces protein-SNO formation and positively regulates SA-induced defense responses [123]. Recent findings suggest that the activation of the GSNOR1 enzyme by GSH results in the alleviation of inhibition of *ICS1* expression in the presence of H_2_O_2_ [124]. In contrast, inactive GSNOR1 results in the buildup of GSNO, thereby causing the inhibition of *ICS1* expression. Additionally, GSNOR1 undergoes posttranslational activation through direct denitrosylation in a GSH-dependent manner. A reduction in protein-SNO formation results in intact protein-SH, and this process results in enhanced *ICS1* expression and SA accumulation (Figure 6). GSH can also react directly with protein-SNOs to form protein-SH. The accumulation of SA triggers the expression of defense genes by inducing conformational changes in the NON-EXPRESSOR OF PATHOGENESIS-RELATED 1 protein (NPR1). In plants that have not been challenged by pathogens, NPR1 remains in the cytoplasm as an inactive oligomer, sustained by redox-sensitive intermolecular disulfide bonds. The S-nitrosylation of the Cys156 residues of NPR1 is crucial for preserving its oligomeric form. Upon a pathogen challenge, alterations in the redox status of plant cells prompt the reduction of cysteine residues in NPR1, catalyzed by thioredoxins (TRXs), causing NPR1 monomers to dissociate from the oligomeric complex [125]. Conversely, the S-nitrosylation of NPR1 monomers by GSNO promotes its oligomerization. Studies have demonstrated that Arabidopsis NPR1 acts as a receptor for SA, and the interaction between SA and NPR1 is essential for the monomerization and subsequent activation of NPR1 [126]. The activated monomer of NPR1 is translocated from the cytoplasm into the nucleus, where it exerts its transcriptional function in SA-related genes. GSNO treatment facilitates the nuclear translocation and accumulation of NPR1 and its interaction with TGA transcription factors. It has also been shown that the S-nitrosylation of TGA1 enhances its DNA-binding activity in NPR1’s presence [127] (Figure 6).

Total S deprivation in Arabidopsis results in SA accumulation and the further activation of the SA signaling pathway via the function of NPR1 [128]. The interplay between S metabolism and SA signaling also plays a major role in the mitigation of stress effects in salinity-exposed plants [129]. In maize seedlings, irrigating the roots with SA increased L-cysteine desulfhydrase activity, which, in turn, led to the accumulation of H_2_S, ultimately enhancing heat tolerance [130]. Likewise, SA stimulated the activity of L-cysteine desulfhydrase in Arabidopsis, leading to an increase in H_2_S production and, ultimately, to higher resistance to cadmium stress [131]. The expression levels of *PAD4* and *EDS1*, which are associated with SA biosynthesis, were increased in Arabidopsis plants exposed to elevated H_2_S concentrations. Conversely, their expression was diminished in plants with lower levels of H_2_S [132]. Recent evidence strongly suggests that SA plays a significant role in plant defense against various environmental stresses, such as heat and drought [133]. It was shown that the application of SO_2_ to maize seedlings before heat stress is beneficial for mitigating the deleterious effects. SO_2_ pretreatment serves to activate SA synthesis, through which plants cope with stress, mainly by activating the antioxidant defense system [134]. On the other hand, the SA treatment of wheat seedlings exposed to heat stress had a stimulating effect on S assimilation by increasing ATP-sulfurylase activity [135]. S and SA collectively reduced the negative effects of arsenic on *Brassica napus* through the tempering of oxidative stress and the enhancement of photosynthetic capability [136]. The precise mechanisms of SA-mediated stress responses are still under investigation, and additional research is required to fully grasp its complexity.

## 10. Strigolactone Crosstalk with S Metabolism

Strigolactones (SLs) are carotenoid-derived plant hormones and signaling molecules that are fundamental for the recognition of the plant by symbiotic fungi but also regulate physiological processes to adapt plant architecture to nutrient availability [137,138]. In rice, SL production is induced in response to S deficiency and inhibits shoot branching while, at the same time, accelerating leaf senescence [139,140]. The primary factor behind this was found to be the upregulation of a gene responsible for SL biosynthesis, *DWARF27 (D27)*, while the expression of other genes involved in SL biosynthesis remained unchanged [130]. These findings indicate that D27 might have a significant impact on efficient sulfur acquisition through arbuscular mycorrhizal fungi, as they were shown to provide sulfate ions in addition to nitrogen and phosphate [141]. Using a split-root assay in rice, it was shown that shoots, not roots, recognize S deficiency, which induces SL production in the roots [142]. However, the shoot-derived signal for SL production is unknown. In a maize mutant deficient in SL biosynthesis (*zmccd8*), the altered expression of sulfate transporters genes was detected, suggesting that sulfate uptake and translocation are also controlled by SL [143]. 

## 11. Conclusions

**Figure 7:**
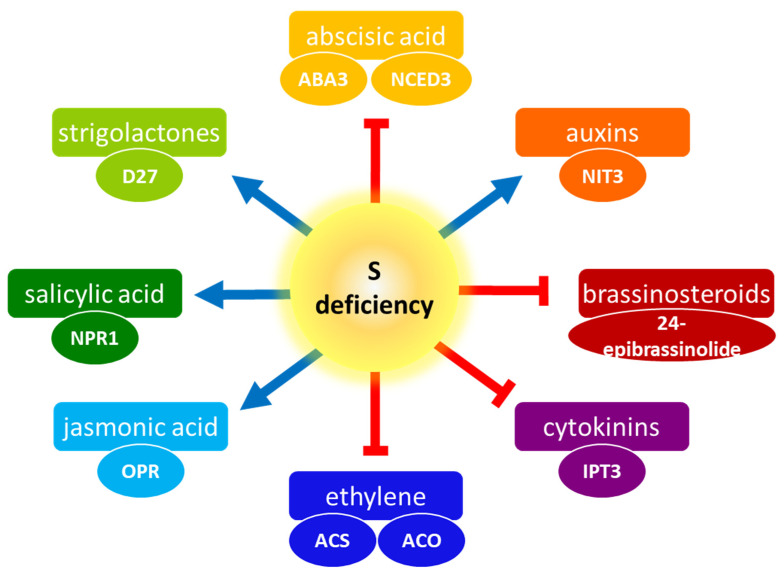
The influence of inadequate sulfur (S) nutrition on phytohormones. The known enzyme/transcription factor activities affected by S deficiency are marked in ovals below each phytohormone. The abbreviations are as follows: sulfurylase ABA DEFICIENT 3 (ABA3), 9-cis-epoxycarotenoid dioxygenase 3 (NCED3), nitrilase 3(NIT3), isopentenyltransferase 3 (IPT3), ACC synthase (ACS), ACC oxidase (ACO), 12-oxophytodienoate reductase (OPR), NON-EXPRESSOR OF PATHOGENESIS-RELATED 1 protein (NPR1), and DWARF27 (D27). Positive and negative impacts on phytohormone levels are indicated by blue and red lines, respectively. Refer to the text for a detailed description.

S nutrition plays a crucial role in the growth and development of plants, influencing their response to both biotic and abiotic stresses, as well as the yield and quality of crops. The gradual emergence of S deficiency in soils has become a significant factor limiting plant growth and crop yields. Despite its importance, there is currently insufficient research on plant S nutrition compared to other macronutrient elements. A comprehensive understanding of the regulation of S nutrition in plants, especially the role of phytohormones in this process, is still elusive. Currently, phytohormones are recognized as pivotal targets for enhancing both plant productivity and stress tolerance, exerting a significant influence on the yield and quality of crop plants. The current review underscores the significance of S in the regulation of phytohormone-mediated responses. It appears that S is a vital requirement for phytohormones to perform optimally. A brief summary of the influence of S deficiency on plant hormones is depicted in Figure 7. Further exploration into the molecular genetics of these aspects would be intriguing and valuable for a deeper understanding. It is also necessary to examine the influence of S availability on yet other important phytohormones—polyamines and peptide hormones—or the effect of these phytohormones on S metabolism, as this field is understudied. Therefore, there is a pressing need for more extensive research to unravel the mechanisms behind the plant’s response to S-deficiency stress, including phytohormonal signaling. Such insights will serve as a foundation for enhancing S utilization efficiency in crops.
